# Near-Infrared-II Bioimaging for *in Vivo* Quantitative Analysis

**DOI:** 10.3389/fchem.2021.763495

**Published:** 2021-11-15

**Authors:** Sha Yang, Xiaofeng Tan, Li Tang, Qinglai Yang

**Affiliations:** ^1^ The First Affiliated Hospital and Center for Molecular Imaging Probe, Hunan Province Key Laboratory of Tumor Cellular and Molecular Pathology, Cancer Research Institute, Hengyang Medical School, University of South China, Hengyang, China; ^2^ Department of Pathology and Tumor Pathology Research Group, Xiangnan University, Chenzhou, China

**Keywords:** NIR-II bioimaging, *In vivo* quantitative analysis, ratiometric, fluorescence, photoacoustic, lifetime

## Abstract

Near-Infrared-II (NIR-II) bioimaging is a newly emerging visualization modality in real-time investigations of biological processes research. Owning to advances in reducing photon scattering and low tissue autofluorescence levels in NIR-II region (1,000–1700 nm), NIR-II bioimaging affords high resolution with increasing tissue penetration depth, and it shows greater application potential for *in vivo* detection to obtain more detailed qualitative and quantitative parameters. Herein, this review summarizes recent progresses made on NIR-II bioimaging for quantitative analysis. These emergences of various NIR-II fluorescence, photoacoustic (PA), luminescence lifetime imaging probes and their quantitative analysis applications are comprehensively discussed, and perspectives on potential challenges facing in this direction are also raised.

## Introduction

Recent studies have showed that bioimaging in the second near-infrared window (NIR-II, 1,000–1700 nm) can be reduced by photon scattering and tissue autofluorescence, leading to a better spatial-temporal resolution and deeper tissue penetration than that of conventional UV-Vis-NIR window (200–1,000 nm) (Smith et al., 2009; Welsher et al., 2009; Hong et al., 2014; Diao et al., 2015a). NIR-II bioimaging holds tremendous potential for real-time, non-invasive, and multi-dimensional investigations (Hong et al., 2017; Fan et al., 2018; Fu et al., 2021) *in vivo* biological processes. In recent years, to further understand the mechanisms of biological processes, especially for diseases related systems, researchers have spared no efforts to produce superior NIR-II contrast agents (Cai et al., 2019; Zhu et al., 2019; Yang Q. et al., 2021; Lei and Zhang, 2021), and to develop advanced NIR-II bioimaging technologies (Liu P. et al., 2020; Luo et al., 2020) (Zhao et al., 2020).

The desirable NIR-II contrast agents demand high performance of optical properties, biocompatibility, activatability, and chemical modification. They have gone through the development process from inorganic to organic materials as well as from polymer macromolecules to small molecules. Inorganic materials have been developed, for example, carbon nanotubes (Robinson et al., 2012; Diao et al., 2015b; Hong and Dai, 2016), quantum dots (QDs) (Dong et al., 2013; Zhu et al., 2013; Liu P. et al., 2020; Liu H. et al., 2020; Yang H. et al., 2021), and rare Earth nanoparticles (Naczynski et al., 2013; Villa et al., 2014; Kong et al., 2019), have been employed as NIR-II fluorophores, and NIR-II organic materials have also been synthesized, such as donor-acceptor (D-A) conjugate polymer or molecule (Antaris et al., 2016; Zhang et al., 2016; Jiang et al., 2019; Gao et al., 2020), cyanine/polymethine/bodipy molecule (Zhu et al., 2018a; Shi et al., 2018; Lei et al., 2019; Godard et al., 2020; Bian et al., 2021; Cosco et al., 2021), and organometallic complex (Yang et al., 2018; Shen et al., 2021). Due to the improvement of NIR-II contrast agents and traditional UV-Vis-NIR bioimaging technologies (Fluorescence, Photoacoustic, Fluorescence-Lifetime, etc.), NIR-II bioimaging technology has gained rapid development recently, providing versatile platforms for *in vivo* bioimaging from macroscopic of tissues (Welsher et al., 2011; Hong et al., 2012; Yang et al., 2018; Kong et al., 2019; Zheng et al., 2019), organs (Zhu et al., 2018b; Kong et al., 2019) and tumors (Zhu et al., 2017; Sheng et al., 2018) to micron-level of vessels (Liu et al., 2020c), lymph (Antaris et al., 2016; Tian et al., 2020), and cells (Chen et al., 2018; Liao et al., 2021)and from two-dimensional (2D) to three-dimensional (3D) with a greater degree of clarity, such as the NIR-II surgical navigation system (Suo et al., 2019; Yu et al., 2019), NIR-II confocal/spinning-disc confocal microscope (Zhu et al., 2018b; Zubkovs et al., 2018), NIR-II light sheet microscope (Wang F. et al., 2019; Wang F. et al., 2021), NIR-II two-photon/multiphoton microscope (Qi et al., 2018), and NIR-II fluorescence lifetime microscope (Yu et al., 2019).

**FIGURE 1:**
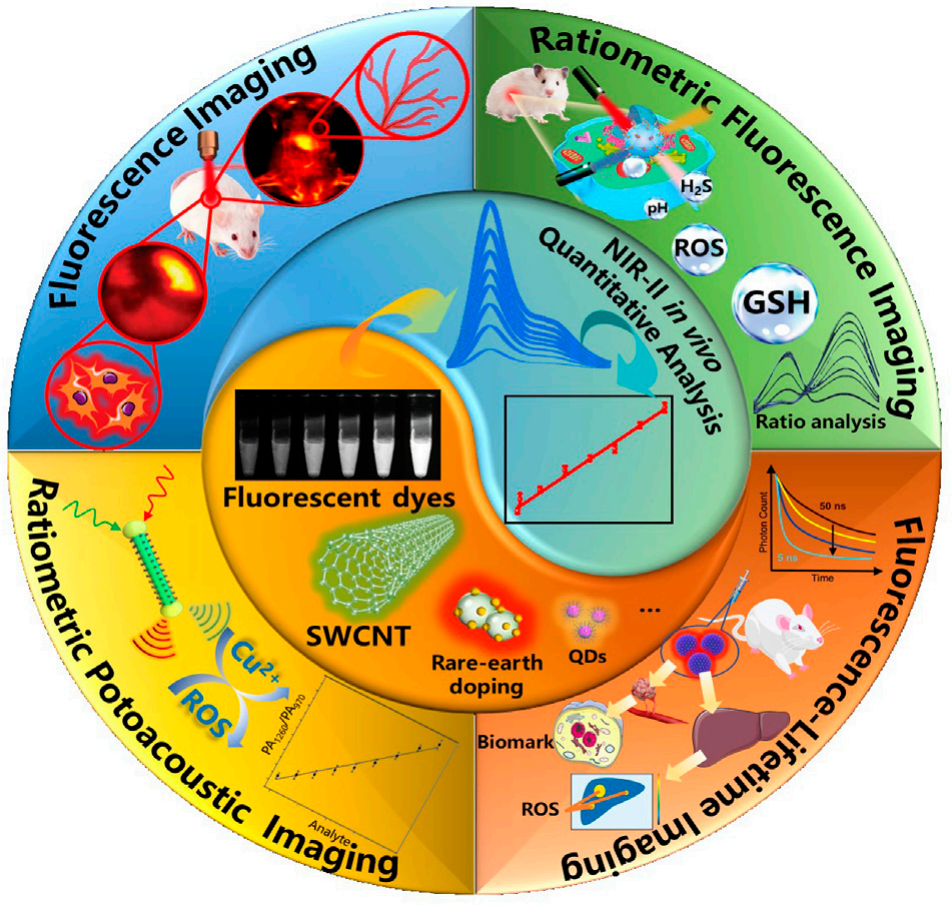
Schematic illustration of NIR-II bioimaging for *in vivo* quantitative analysis.

Traditional UV-Vis-NIR bioimaging technologies have been applied in a variety of *in vitro* quantitative analysis, but such tissue penetration depth is generally impractical *in vivo*. Fortunately, the combination of superior NIR-II contrasts and advanced NIR-II bioimaging technologies can provide more detailed image parameters, which is suitable for *in vivo* quantitative analysis to understand biological process. In this review, we have summarized various types of NIR-II probes for *in vivo* quantitative analysis applications, including NIR-II fluorescence imaging (Welsher et al., 2011; Wang F. et al., 2019; Yu et al., 2019; Liu et al., 2020c; Tian et al., 2020), NIR-II ratiometric fluorescence/photoacoustic imaging (Ye et al., 2020; Wang et al., 2021b; Wang et al., 2021c; Fu et al., 2021), and NIR-II fluorescence-lifetime imaging (Fan et al., 2018; Zhao et al., 2020) for *in vivo* quantitative analysis, and also raised perspectives on potential challenges facing in this direction (Figure 1).

## NIR-II Fluorescence Imaging for *in Vitro* Quantitative Analysis

Compared with NIR-I bioimaging, NIR-II bioimaging has attracted much more attentions *in vivo* imaging due to its deeper penetration depth (∼cm) and superior imaging resolutions (∼μm) (Monici, 2005; Welsher et al., 2009; Diao et al., 2015a; Cai et al., 2019), especially suitable for *in situ* imaging of tissues/organs in small animal models for fundamental and preclinical researches. Therefore, NIR-II bioimaging can also be suitable for most of the applications used by UV-Vis-NIR bioimaging in *in vitro* examination.

### *In Vitro* Quantification for NIR-II Fluorescent Materials

**FIGURE 2:**
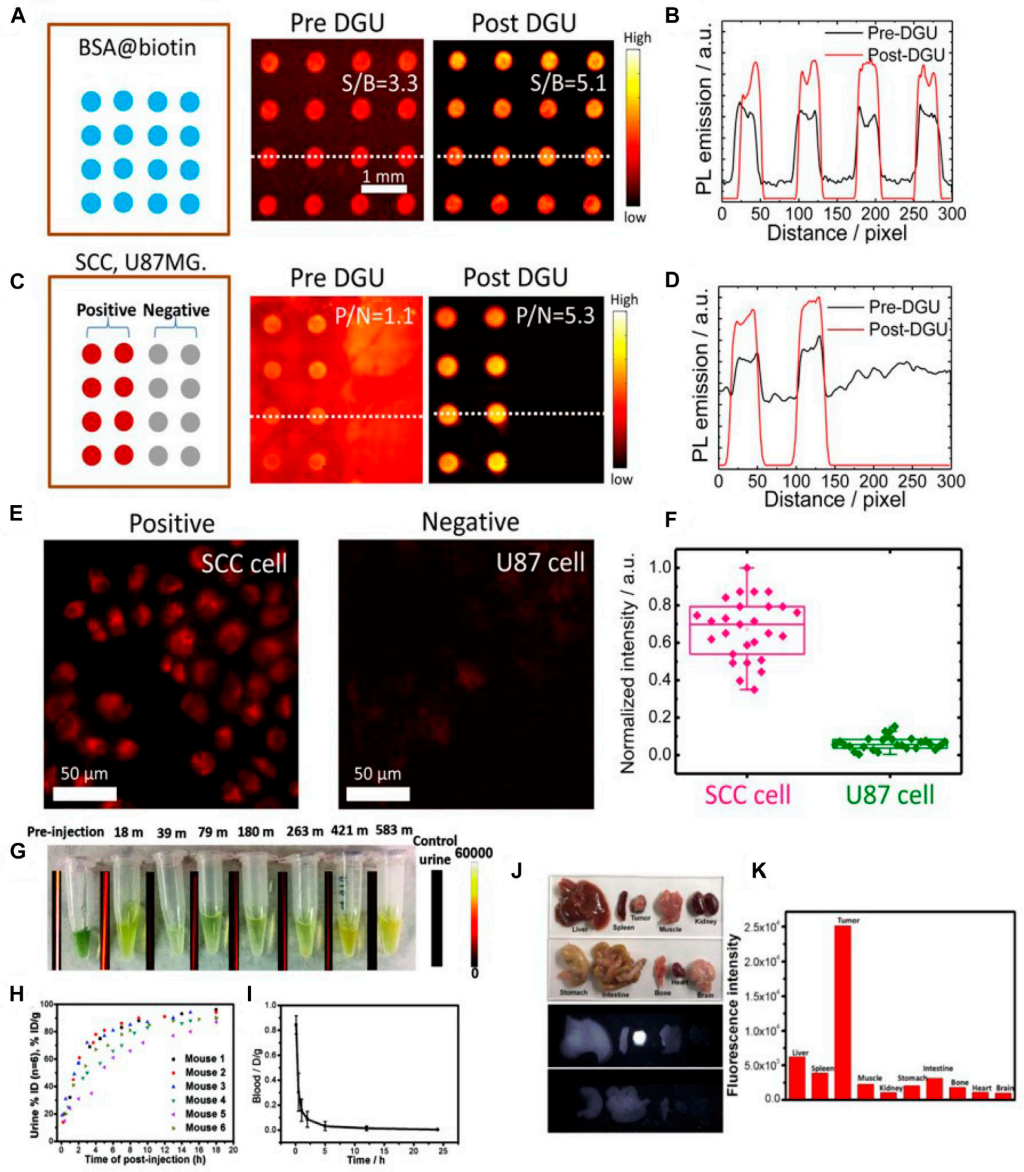
The microarray assay screening method developed to test the conjugates. **(A)** Assay analysis of DGU fraction of SA@IR-FGP. **(B)** Cross-sectional intensity profile of the spot signal. **(C,D)** Assay analysis of the DGU fractions of Erb@IR-FGP. SCC and U87 cell lysates were printed on plasmonic fluorescence-enhancing gold slides for testing the conjugate Erb@IR-FGP. **(E)** Targeted cell staining by Erb@IR-FGP (SCC and U87 cell lines as positive and negative controls) scanned by a home-built microscope with 785 nm excitation and a 1,050 nm long-pass emission filter. **(F)** PL intensity statistic of SCC and U87 cells (reproduced from (Zhu et al., 2017) with permission from National Academy of Sciences.) **(G)** Collections of urine of mice injected with NIR-II fluorophore IR-BGP6 and their corresponding fluorescence images. **(H)** Quantification of renal excretion of IR-BGP6 by calculation of its amount in urine. ≈ 91% IR-BGP6 can be renally excreted within the first 10 h p. i. **(I)** The blood circulation behavior of IR-BGP6. The fast-renal excretion of IR-BGP6 corresponded to the short blood circulation half-life time (≈24 min). **(J)** Major organs and tumors taken from mice injected with anti-PD-L1-BGP6 at 24 p. i. and their corresponding fluorescence imaging in the NIR-II window (>1000 nm). **(K)** Quantification of biodistribution of anti-PD-L1-BGP6 (reproduced from (Wan et al., 2018) with permission from Wiley-VCH Verlag GmbH and Co. KGaA, Weinheim).

Zhu et al. have used a home-built microarray assay screening method and a home-built microscope setup to evaluate the activity of the bioconjugate between NIR-II fluorophore IR-FGP and protein (IR-FGP@protein) in a fluorescence intensity quantitative manner (Zhu et al., 2017). Incubated with IR-FGP@protein or Erbitux@IR-FGP conjugate, the spots showed bright fluorescence emission. The selective binding between serum albumin (SA) and biotin or Erbitux (Erb) and epidermal growth factor receptor (EGFR) proved the IR-FGP molecular selectivity (Figures 2A–C). The cross-sectional line outlines of the fluorescence intensity at the two detection points clearly indicated that the signal-to-background ratio (SBR) had increased significantly after density gradient ultracentrifugation (DGU) purification (Figure 2B). After DGU separation, Erb@IR-FGP conjugates showed increased microarray assay positive/negative (P/N) ratios (EGFR^+^ and EGFR^−^) from 1.1 (pre-DGU) to 5.3 (Figure 2D). Molecular imaging of cells labeled with Erb@IR-FGP conjugate showed that the fluorescence from EGFR^+^ SCC cells was stronger than that of EGFR-U87MG cells (Figures 2E,F), which also showed an excellent selectivity.

Wan et al. have used the NIR-II *in vitro* fluorescence quantitative method to determine the pharmacokinetics of NIR-II fluorophore IR-BGP6 in normal mice, and also to survey the biodistribution of the conjugate anti-PD-L1-BGP6 in MC38 tumor-bearing mice (Wan et al., 2018). Attribute to the avoidance of NIR-II fluorescence in body fluid’s autofluorescence, the renal excretion kinetics was obtained through collecting urine with the accumulated fluorescence intensity in the NIR-II window after 24 h (Figure 2G). For a better observation, ≈91% of IR-BGP6 was excreted through urine from most mice within the first 10 h p. i. (Figure 2H). Similarly, the examination for blood half-life time, the percentage of IR-BGP6 in blood was ≈84.3% ID g^−1^at 6 min p. i. and declined to ≈15.8% ID g^−1^ at 1 h p. i, showing a blood half-life time of ≈24 min, in consistent with the fast renal excretion kinetics (Figure 2I). Quantification of the imaging of *in vitro* organs taken from mice inoculated with anti-PD-L1-BGP6, the biodistribution at 24 h p. i. showed that most anti-PD-L1-BGP6 accumulated within tumors in contrast to the relatively low remaining within other major organs (Figures 2J,K). Although these methods are derived from the traditional UV-Vis-NIR technology, the uniqueness advantages of NIR-II *in vitro* quantitative analysis make it indispensable in the NIR-II materials research.

### *In Vitro* Quantification for Point of Care Testing

**FIGURE 3:**
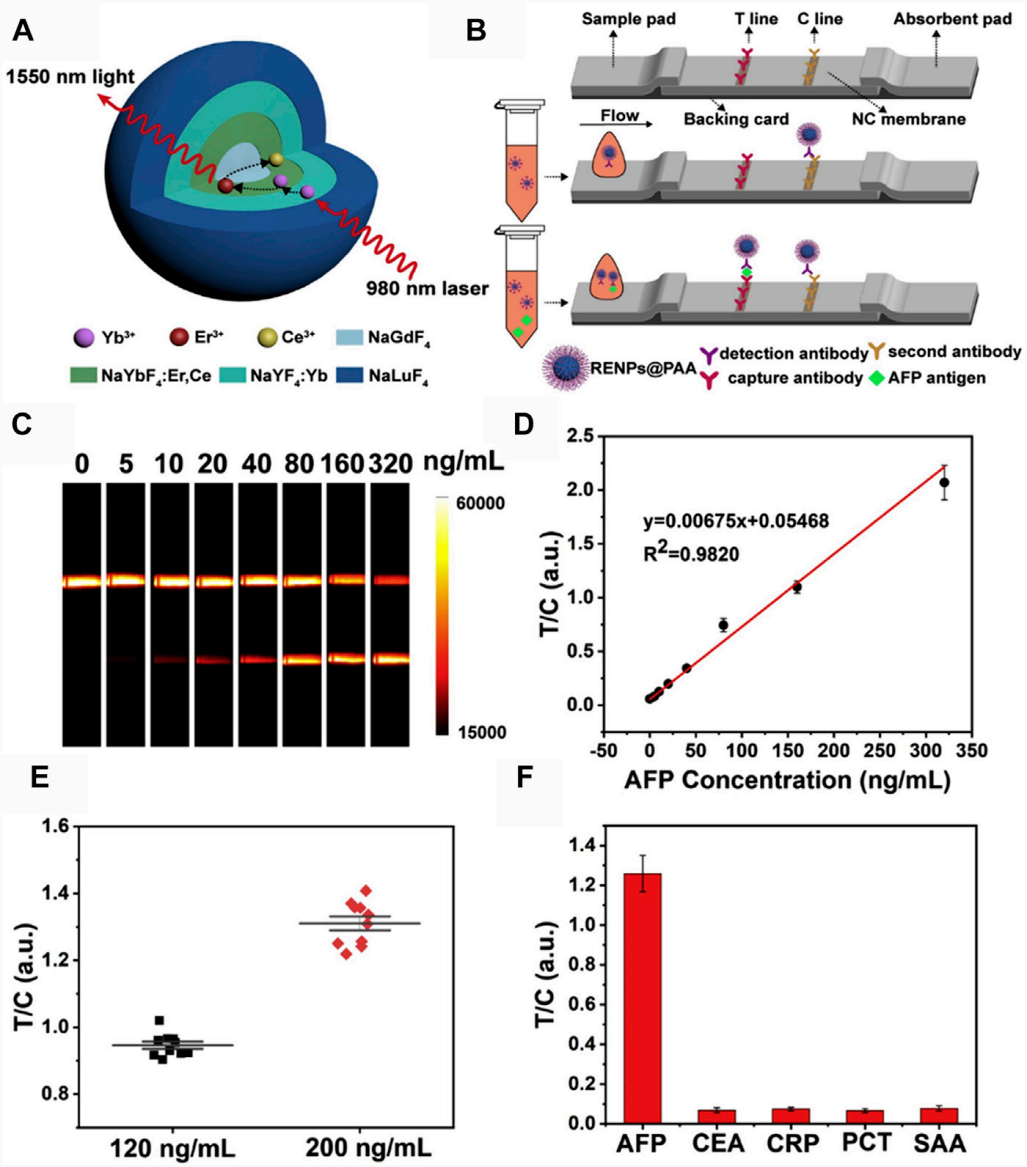
NIR-II rare-earth nanoparticles for a lateral flow immunoassay in hemolysis **(A)** Structural design of the Yb, Er/Ce co-doped NIR-II rare-earth nanoparticles (Er,Ce-CSSS) with 1,550 nm emission under 980 nm excitation. **(B)** Schematic illustration of NIR-II rare-earth nanoparticles based lateral flow immunoassay platform (NIR-II LFA). **(C)** Strip images of AFP standards spiked in hemolysis by NIR-II LFA test. Exposure time was 100 ms. **(D)** The calibration curve for AFP detection in hemolysis. Error bars represent the stand deviations calculated from three separate experiments. **(E)** Reproducibility of NIR-II LFA for AFP detection at the concentrations of 120 ng/ml and 200 ng/ml. **(F)** Specificity test of NIR-II LFA using different biomarkers. The concentrations of CEA, CRP, PCT and SAA were 10 times higher than that of AFP (reproduced from (Li et al., 2021) with permission from Elsevier B.V).

Point of care testing (POCT), known as near-patient testing, includes rapid response to facilitate pathological decisions, as well as ease of use. Fluorescent lateral flow immunoassay (LFA) is a popular diagnostic tool used in POCT. However, body fluid detection using LFA with fluorescent labels is still facing the challenges of autofluorescence interference, light absorption, and scattering in UV-Vis-NIR region. It is clear that NIR-II fluorescence can well be done to solve this problem. Li et al. established an LFA platform using Yb, Er, Ce co-doped core-shell-shell-shell nanoparticles (Er, Ce-CSSS) with 1,550 nm emission as fluorescent labels (Figure 3A) (Li et al., 2021). Er,Ce-CSSS-antibody probe was pre-mixed with AFP antigen, then the complex was trapped by the capture antibody on test (T) line, while the excess Er,Ce-CSSS-antibody probe continued to move until it bound with the second antibody on control (C) line. Then, the strips were placed into the designed imaging system to collect the luminescence signals (Figure 3B). To evaluate the quantitative detection capability of this NIR-II LFA, a calibration curve for AFP detection in hemolysis was established by preparing a series of AFP standard in negative hemolytic samples (Figures 3C,D). As the calibration curve showed that the limit of detection (LOD) of AFP in hemolysis was determined to be 1.00 ng/ml, which was 20 times lower than the clinical cut-off value of 20 ng/ml. To verify the high reliability of NIR-II LFA platform, the assay reproducibility was examined by testing 10 strips with AFP samples at the concentration of 120 ng/ml and 200 ng/ml, respectively. The T/C ratios were highly centralized with a CV of 3.6 and 4.9%, respectively (Figure 3E). To further study the specificity of NIR-II LFA, AFP as well as general clinical biomarkers that were usually detected in blood, such as carcino-embryonic antigen (CEA), C-reactive protein (CRP), procalcitonin (PCT), and serum amyloid protein A (SAA) (Figure 3F). The T/C ratio of AFP was markedly higher than that of other analytes, indicating that NIR-II LFA is a specific POCT method. As these advantages of the above-mentioned in NIR-II materials researches and POCT, NIR-II *in vitro* quantitative methods are suitable for most application scenarios of the traditional UV-Vis-NIR methods, and form supplements for its deficiencies.

## NIR-II Fluorescence Imaging for *in Vivo* Quantitative Analysis

A goal of ideal *in vivo* bioimaging technology is to achieve high-fidelity and real-time quantitative analysis for *in situ* visualization monitoring. *In situ* quantitative analysis *in vivo* can provide the dynamic information of biological processes in real time, which is significance to explore the pathogenesis of diseases (Wan et al., 2019; Liu P. et al., 2020). Compared with the clinical *in vivo* bioimaging technology, such as PET-CT, MRI, and ultrasound imaging, the tissue penetration depth of NIR-II bioimaging is only centimeter level, but it has the advantages of high temporal resolutions (∼ms) for real-time monitoring and high resolutions (∼μm) for micro-quantitative detections, which can be performed *in situ* quantitative analysis *in vivo* of small animal models for fundamental researches, and give opportunities to achieve the above goal. As traditional Vis-NIR bioimaging technologies, the basic primary function of NIR-II *in vivo* bioimaging is to distinguish the pathological targets from surrounding tissues (signal-to-noise ratio: S/N value) by the fluorescence quantification. With the development of NIR-II probes and instrument technologies as well as the powerful data processing capabilities by computational evaluation, more quantitative information can be obtained through NIR-II *in vivo* bioimaging.

### *In Vivo* Quantitative Analysis for Vasculatures

**FIGURE 4:**
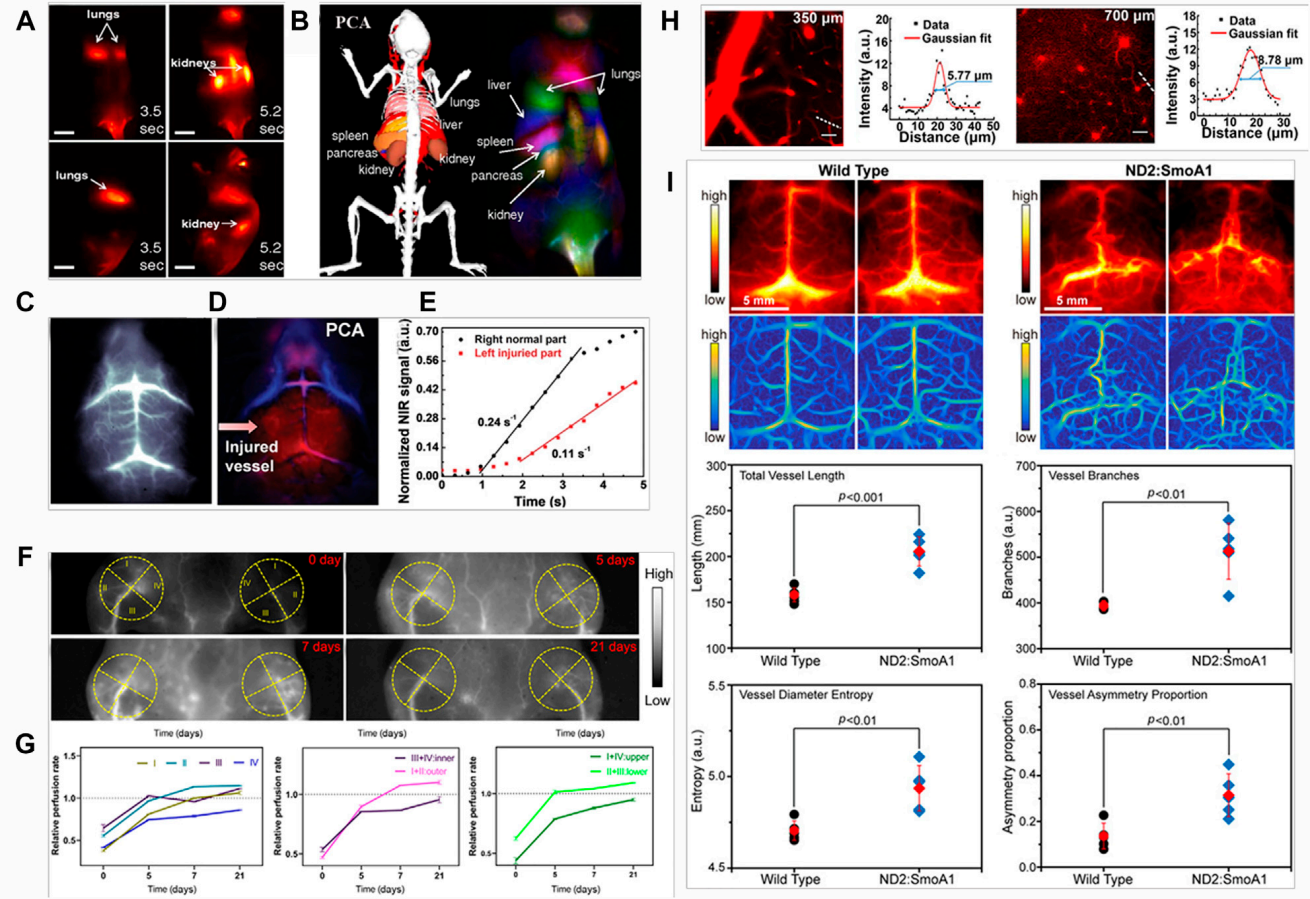
Examples of NIR-II fluorescence imaging for *in vivo* quantitative analysis. **(A)** Frames from video imaging of mice injected with NIR-II fluorophore SWNTs. **(B)** Dynamic contrast-enhanced imaging with SWNTs through PCA. This feature is not observable in the raw time-course images (reproduced from (Welsher et al., 2011)with permission from National Academy of Sciences.). **(C)** Dynamic brain imaging of stroke mouse. **(D)** PCA overlaid images with arterial (red) and venous (blue) vessels of stroke mouse, indicating more significant injured signal in the left brain. **(E)** Blood perfusion of stroke mouse, showing slower blood flow in the injured left brain than the health right brain. Errors bars indicate the standard deviation of each group (reproduced from (Liu et al., 2019) with permission from WILEY-VCH Verlag GmbH and Co. KGaA, Weinheim.). **(F)** NIR-II fluorescence images of hindlimb vessels at 0, 5, 7 and 21 days postischemia. **(G)** The relative perfusion rate of four quadrants, upper and lower quadrants, inner and outer quadrants (reproduced from (Chen et al., 2020)with permission from Tsinghua University Press and Springer-Verlag GmbH Germany, part of Springer Nature). **(H)** *In vivo* NIR-II fluorescence confocal scanning microscopic images of mouse brain vasculature at 350 and 700 μm depth, and the Gaussian fits to the profiles along the capillary vessels indicated by the white-dashed lines (reproduced from (Yu et al., 2019) with permission from Elsevier B.V. and Science China Press.). **(I)** *In vivo* NIR-II fluorescence images **(top panel)** and Hessian-matrix-enhanced images **(bottom panel)** of cerebral vasculature of wild-type C57BL/6 mice and ND2:SmoA1 mice (n = 5). Red data are reported as mean: standard deviation (reproduced from with permission (Liu et al., 2020c)from Wiley-VCH GmbH.).

Welsher et al. have performed *in vivo* imaging of mice during intravenous injection of single-walled carbon nanotubes (SWNTs), using NIR-II *in vivo* imaging setup to observe the process of SWNTs circulate through the lungs and kidneys in several seconds (Figure 4A), and the spleen and liver at slightly later time points (Welsher et al., 2011). Dynamic contrast-enhanced imaging through principal component analysis (PCA) was performed to obtain the anatomical resolution of organs as a function of time post-injection (Figure 4B). Importantly, PCA is a common statistical processing method for compressing high-dimensional data into a lower-dimensional form by choosing only the highest variance components of the dataset, which is a powerful tool for analyzing time related activity (Ringner, 2008; Abdi and Williams, 2010). NIR-II *in vivo* bioimaging combined with PCA analysis has represented a powerful approach to high-resolution optical imaging through deep tissues and been useful for a wide range of applications in biomedical researches. Liu et al. performed NIR-II *in vivo* imaging and PCA analysis in cerebrovascular diseases with an ischemic stroke model (Liu et al., 2019). Brain vessel imaging with NIR-II gold clusters was gathered *via* tail intravenous injection. It could be clearly observed the intensity of neovascularization in the left brain, the injured left brain exhibited more arterial vessels compared with the right brain (Figure 4C). PCA analysis could distinguish that the red and blue vessels are arterial and venous vessels, respectively. It showed the significant red signal (red arrow) in the left brain (Figure 4D). The dynamic blood perfusion was also evaluated by normalizing NIR-II fluorescence signal in the arterial vessels of both the left and right brain, and the results showed that blood perfusion rate of the injured left brain was 0.11 s^−1^, which was two times lower than the normal right brain of 0.24 s^−1^ (Figure 4E).

NIR-II fluorescence quantitative analysis not just allows us to monitor neovascularization of damaged brain tissue after a stroke, more importantly, it shows a great promise in tracking the pathophysiological process of neovascularization dynamically *in vivo*. Chen et al. have studied the mechanism of neovascularization in hindlimb vessels, and analyzed the spatial distribution of neovascularization (Chen et al., 2020). NIR-II fluorescence images of hindlimb vessels at 0, 5, 7, and 21 days postischemia, four quadrants were divided as shown in the yellow dots (Figure 4F). The relative perfusion rates were calculated by fluorescence intensity quantification. The results showed that all quadrant rates increased with time, except for that of III quadrants from 5 to 7 days post-ischemia, and the outer quadrant rate was more than that of the inner quadrant from 5 to 21 days postischemia, the lower quadrant rate was more than that of the upper quadrant in 3 weeks postischemia (Figure 4G). This suggested that the restoration of blood flow perfusion was better in these quadrants, possibly because of the higher volume of newly formed blood vessels. Notably, the relative perfusion rates of III and lower quadrants at 5 days post-ischemia, II, outer and lower quadrants at 7 days postischemia, I, II, III, outer and lower quadrants at 21 days postischemia were more than 1. It indicated that the blood flow perfusion of these quadrants at these time points had exceeded that of pre-ischemia due to neovascularization during 3 weeks postischemia.

In terms of the vasculature research, more quantitative information can be obtained *via* NIR-II *in vivo* bioimaging. Yu et al. have integrated the advantages of NIR-II fluorescence confocal microscopic with high spatio-temporal resolution and NIR-II aggregation-induced emission (AIE) dots with high brightness, high-resolution *in vivo* cerebrovascular imaging of a mouse, the spatial resolution at 350 and 700 μm depth could reach 5.77 and 8.78 μm, respectively (Figure 4H) (Yu et al., 2019). Liu et al. have assessed and quantified the vascular morphology of the transgenic brain tumors with vascular segmentation and quantification algorithm (Liu et al., 2020c). Based on a modified Hessian matrix method (Yang et al., 2014), the Hessian-matrix-enhanced images were generated from the original *in vivo* fluorescence images. By using the extracted enhanced image and identified centerlines, the vascular morphology of transgenic brain tumors in terms of the vessel lengths, vessel branches, and vessel symmetry was quantitatively analyzed, which showed statistically significant differences from the wild type mice (Figure 4I).

### *In Vivo* Quantitative Analysis for Cells Fate

**FIGURE 5:**
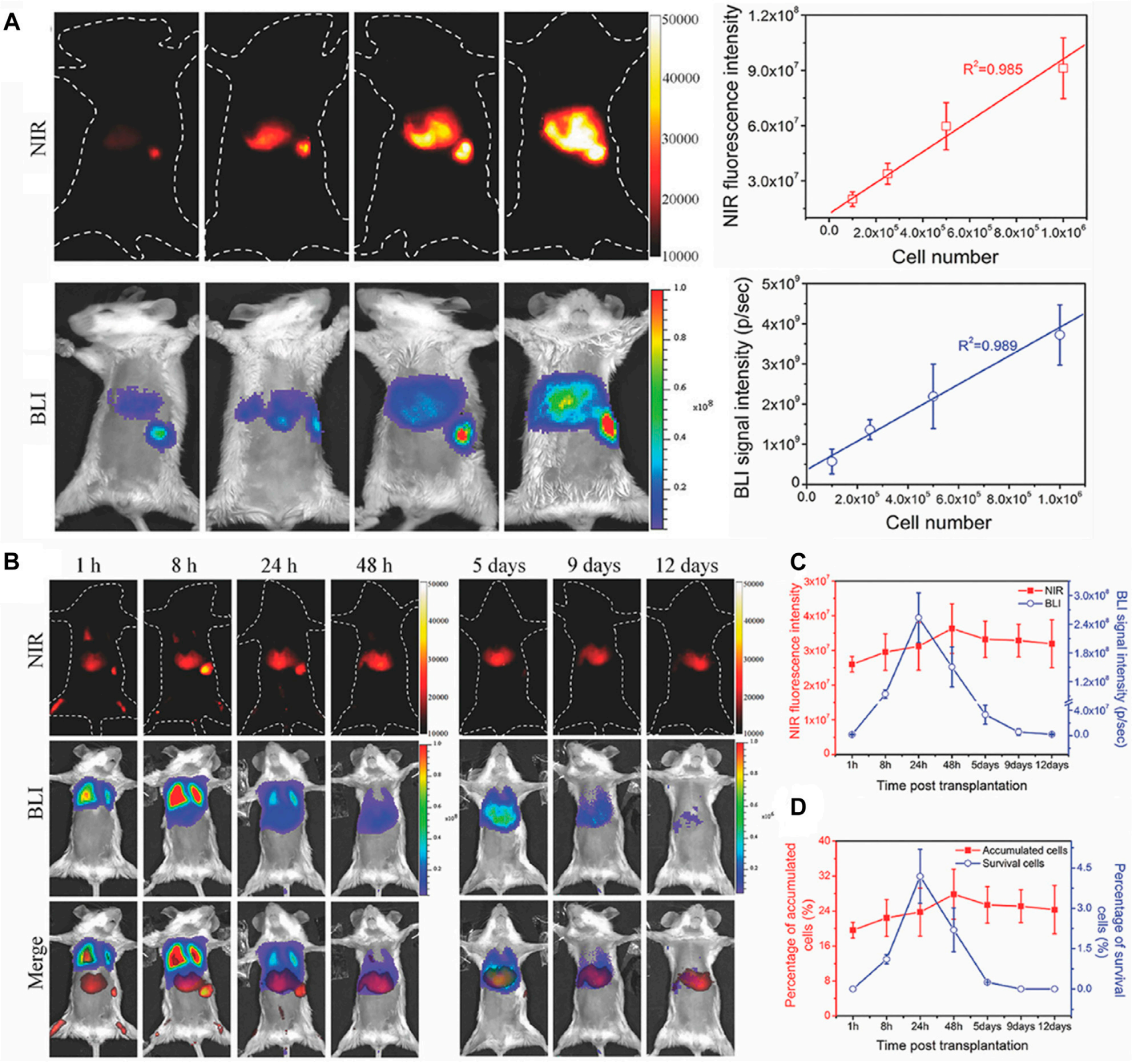
*In vivo* quantitative analysis for cells fate. **(A)** *In vivo* imaging and quantifying of RfLuc and Tat-Ag_2_S QD dual-labeled mMSCs. NIR-II fluorescence images and BLI images of dual-labeled mMSCs after injecting *via* the spleen in mice, and the linear correlation of the cell numbers of transplanted mMSCs with the NIR-II fluorescence intensities and BLI signals in liver and spleen. **(B)** *In vivo* tracking the fate of intravenously transplanted mMSCs for liver regeneration, NIR-II fluorescence images, BLI images, and merged images of mice with acute liver failure. **(C)** Quantitative analyses of the cell accumulation and survival in liver by the total NIR fluorescence intensities of NIRFI and the total photon flux (photons s^−1^) of BLI. **(D)** Quantitative analyses of the accumulation and survival ratios of mMSCs in liver (reproduced from (Chen et al., 2018) with permission from WILEY-VCH Verlag GmbH and Co. KGaA, Weinheim).

In addition to the quantitative research for *in vivo* target tissues and vasculatures, more interestingly, NIR-II bioimaging can also perform quantitative analyses for target cells fate *in vivo*. Chen et al. have developed a dual-labeling strategy to *in situ* visualize the fate of transplanted stem cells *in vivo via* combining the exogenous NIR-II fluorescence imaging (NIRFL) and endogenous red bioluminescence imaging (BLI) (Chen et al., 2018). The NIR-II Ag_2_S QDs were employed to dynamically monitor the trafficking and distribution of all transplanted stem cells *in vivo*, and the BLI of red-emitting firefly luciferase (RfLuc) identified the living stem cells after transplantation *in vivo*. It was found that both the NIRFI and BLI signal intensities were linearly correlated with the amount of dual-labeled mouse mesenchymal stem cells (mMSCs), with an *R* ^2^ of 0.985 and 0.989, respectively (Figure 5A). According to the good linear relationship between the number of dual-labeled mMSCs and the NIRFI or BLI signal intensities, the distribution and viability of intravenously transplanted mMSCs were explored *in vivo* by the combined BLI/NIRFI bioimaging (Figures 5B,C). The amount of accumulated or survived stem cells were quantitatively analyzed after intravenous transplantation, the quantitative results revealed that 32.9% of transplanted mMSCs were accumulated in the lung, and 19.6% of transplanted mMSCs were accumulated in the liver 1 h p.i. (Figure 5D). This facile strategy allows for the quantitative evaluation of cell translocation and viability with a high spatial-temporal resolution.

## NIR-II Ratiometric Fluorescence Imaging for *in Vivo* Quantitatve Analysis

Because of high sensitivity, simplicity and fast response time, the application of fluorescent probes in optical imaging and analytical sensing has attracted great attentions (Wu et al., 2017). However, providing only a single emission to quantify a target analyte with fluorescent probes that fraught with challenges due to a variety of analyte-independent factors, such as environmental factors, that interfere with the analysis (Lee et al., 2015). In contrast, ratiometric fluorescence as a potential method has received particular attentions to overcome the limitations of the current intensity-based probes. This technique depends on the changes in the intensity of two or more emission bands that induced by an analyte, leading to an effective internal referencing which improves the sensitivity of the detection. The self-calibration and the unique opto-physical properties of nanoparticles (NPs) that compensates for environmental factors and eliminates most of the fuzziness (Park et al., 2020), which have made the ratiometric fluorescent probes more sensitive and reliable, resulting in more rational detection of the analytes, such as ROS (Wang et al., 2019b), enzymes (Makukhin et al., 2016; Ma et al., 2018) and signal molecules (Bianchi-Smiraglia et al., 2017; Umezawa et al., 2017; Wang et al., 2017), biological ions (Aron et al., 2016; Narayanaswamy et al., 2019) and pH (Gao et al., 2017; Richardson et al., 2017)*in vivo* or *in vitro.*

Compared with the traditional NIR-I fluorescence imaging, NIR-II fluorescence imaging can reduce light scattering and background autofluorescence interference to the maximum extent and achieve deeper tissue penetration and higher resolution. Therefore, NIR-II ratiometric fluorescent imaging, based on the advantages of NIR-II fluorescent probes and ratiometric fluorescent probes, can provide a better platform for quantitative analyses *in vivo,* such as Reactive oxygen species (ROS), GSH, signal molecules and pH responsive quantitative analyses.

### ROS

ROS is a general term for a class of molecules or ions with active chemical properties and high oxidation activity, including hydrogen peroxide (H_2_O_2_), hydroxyl radical (·OH), peroxynitrate (ONOO^−^), superoxide (O^2-^), singlet oxygen (^1^O_2_) and so on. Excessive ROS can cause damage to some biological macromolecules (such as lipids, nucleic acids and proteins), thereby affecting their normal physiological functions (Trachootham et al., 2009; Brieger et al., 2012). A large number of studies have shown that ROS is closely related to various pathological diseases, such as cancer, atherosclerosis, diabetes, and inflammation (Zhang et al., 2015).

**FIGURE 6:**
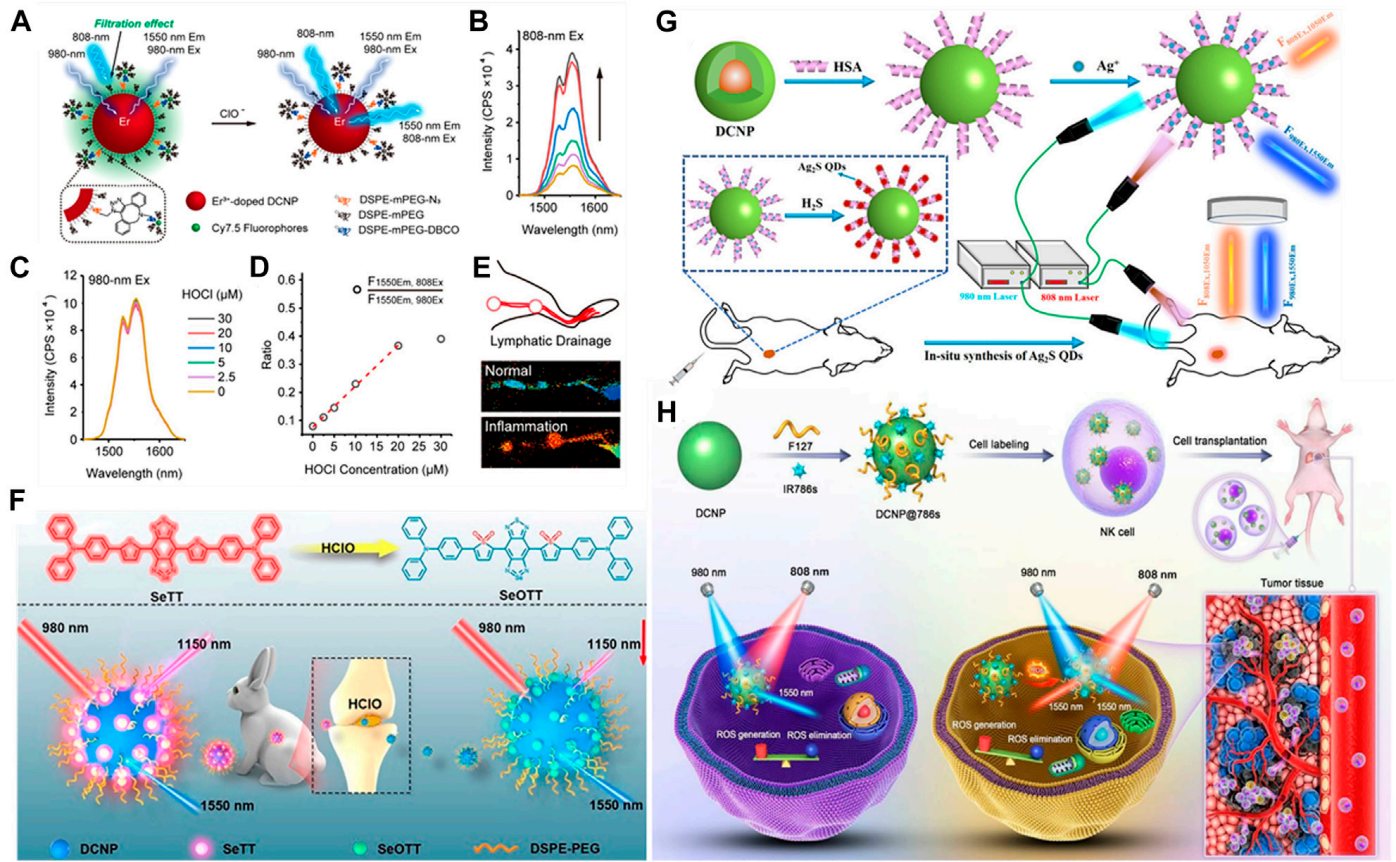
Examples of NIR-II ratiometric fluorescence imaging for *in vivo* quantitative analysis. **(A)** Schematic illustration showing the ratiometric response of NIR-II fluorophore DCNP@Cy7.5 to HClO based on an ACIE mechanism. Er^3+^-doped DCNP: NaEr_x_Y_1_-_x_F_4_@NaYF_4_ (x = 0.05, 0.15, 0.5). **(B,C)** Fluorescence spectra of DCNP@Cy7.5 (10 μM Cy7.5) upon addition of 0–30 μM HClO under 808 and 980 nm excitation, respectively. **(D)** Plot of fluorescence ratio changes as a function of HClO concentration. **(E)** Anatomical structures of lymphatic inflammation in ratiometric channel are clearly observed with a precise resolution (reproduced from (Wang et al., 2019b) with permission from American Chemical Society). **(F)** Schematic illustration showing the ratiometric response of NIR-II fluorophore DCNP@SeTT@PEG: DCNP encapsulated with SeTT and PEG. (reproduced from (Ge et al., 2020) with permission from American Chemical Society). **(G)** Schematic Illustration of the Design of NIR-II Ratiometric FL Nanoprobe for the Detection of H_2_S in Colon Cancer Tissues *in vivo*. (reproduced from (Wang et al., 2021c)with permission from American Chemical Society). **(H)** Schematic illustration of DCNP@786s with ratiometric NIR-II fluorescent signal for tracking NK cell viability *in vivo*. (reproduced from (Liao et al., 2021) with permission from WILEY-VCH).

For a NIR-II organic dye and rare Earth nanoparticles (LnNPs) based ratiometric sensing platform, the detection of target species mainly relies on the measurement of the relative intensity ratio change between the fluorescent dyes and nanoparticles. The representative application is to use a specific organic dye that can chromatically respond to the target analytes as energy acceptor or quencher. Liu et al. have synthesized a new type of LnNPs (NaErF_4_:Ho^3+^@NaYF_4_) with three emission bands (1,180, 980, 650 nm). By encapsulating NaErF_4_:Ho^3+^@NaYF_4_ with IR1061 and Fe^2+^ into a single nanoparticle, a NIR-II ratiometric fluorescence probe (I_980_/I_1180_) was synthesized (Liu et al., 2018). The fluorescence quenching of 980 nm was caused by the strong absorption of IR1061 at 800–1100 nm, and the IR1061 dye was decomposed by the localized OH radicals produced by Fe^2+^ reacted with H_2_O_2_. Subsequently, the 980 nm emission (I_980_) of LnNPs gradually recovered, at the same time, the emission intensity of 1,180 nm (I_1180_) kept almost unchanged. With the excellent characteristics of ratiometric I_980_/I_1180_, the concentration of H_2_O_2_ was measured *in vivo*. In another work, Wang et al. have developed a NIR-II ratiometric fluorescence probe that is an erbium-doped NaYF_4_@NaYF_4_@Cy7.5 composite (DCNP@Cy7.5) (Figure 6A) (Wang et al., 2019b). As expected, the absorbance of DCNP@Cy7.5 at 808 nm would gradually decrease with the titration of 0–30 μM hypochlorous acid (HClO), while the fluorescence of 1,550 nm would gradually recover under the decreasing 808 nm excitation (Figure 6B). With the constant fluorescence excited by 980 nm light as the reference signal (Figure 6C), the fluorescence ratio (F_1550Em,808Ex_/F_1550Em,980Ex_) increased linearly with the concentration of HClO in the range of 0–20 μM (the detection limit:0.5 μM) (Figure 6D). Lymphatic drainage in the hindlimb of mice consists of two lymph nodes and two connective lymph vessels were clearly observed with a precise resolution (Figure 6E). Recently, Cao et al. have constructed a NIR-II ratiometric sensing platform based on cyanine dye (Cy925) and NaYbF_4_:Er@NaYF_4_:Yb@NaYF_4_:Nd nanoparticles (Er-CSSNPs) for the detection of HClO (Cao C. et al., 2019). The Cy925 acted as the HClO sensing component with activatable emission signals at 925 nm wavelength, and the emission of Er-CSSNPs at 1,525 nm wavelength was used as the internal reference. The ratiometric nanoprobe relied on the ratio of aforementioned two separated emission peaks (I_925 nm_/I_1525 nm_), which has been verified to be highly sensitive and selective to HClO *in vivo*.

In addition, Ge et al. have designed a novel activated NIR-II fluorescence molecule (SeTT), which was rapid and specific response to hypochlorous acid (HClO) (Ge et al., 2020). In order to obtain a NIR-II ratiometric probe (DCNP@SeTT), SeTT was coated on the surface of Er^3+^-doped down conversion nanoparticle (DCNP). Under the excitation of 980 nm laser, the ratiometric fluorescence signals of SeTT at 1,150 nm and DCNP at 1,550 nm (I_1150_/I_1550_) was linear relationship with HClO level (the detection limit:0.4 μM). The ratiometric nanoprobe successfully studied the concentration of HClO in the process of tumor progression, visualization of anatomical structures of the peritoneal cavity in inflammatory mice model, and quantitatively detected the concentration of HClO in rabbit osteoarthritis model, obtaining rapid response and high selectivity for detection of HClO (Figure 6F).

Interestingly, some researchers have utilized ROS-response for *in vivo* quantitative analyses for cells fate, for instance, monitoring natural killer cell (NK) cells during adoptive NK cell-based immunotherapy (Guillerey et al., 2016). In NK cells preclinical and clinical studies (Alvarez-Breckenridge et al., 2012), it is still an urgent need to track cell activity to monitor the engraftment efficiency and ultimate fate of NK cells, as well as to evaluate its efficacy and safety during adoptive NK cell immunotherapy. Liao et al. have developed a NIR-II ratiometric fluorescence imaging nanoreporter for real-time quantitative tracking of adoptive NK cells activity *in vivo* (Liao et al., 2021). As a proof of this concept, the nanoreporter consists of DCNP coated with IR786s (a broad-spectrum ROS sensitive probe) for labeling NK cells and NIR-II imaging in a model of orthotopic hepatocellular carcinoma (HCC) *in situ*. Once cells death, excessive ROS was occurred in the tumor microenvironment (TME), leading to the increase of NIR-II fluorescence signal (1,550 nm) excited by 808 nm laser (F_1550Em,808Ex_), and the 1550 nm (F_1550Em,980Ex_) NIR-II fluorescence signal excited by 980 nm was used as a reference (Figure 6H). By using this ratiometric response signal (Ratio = F_1550Em,980Ex_/F_1550Em,808Ex_), they evaluated the production of intracellular excess ROS and tracked the cell activity *in vivo* for adoptive NK cell-based immunotherapy. This method has a great potential in monitoring the efficiency and safety of NK cell implantation *in vivo* and accelerating the clinical outcome of adoptive NK cell immunotherapy.

### GSH

Glutathione (GSH) is the most abundant endogenous antioxidant, and plays an important role in maintaining the balance of redox state in biological system (Chu et al., 2017). Compared with normal tissue cells, the concentration of GSH in cancer cells was much higher, up to 2–10 mM, which was about 1,000 times higher than normal tissues/cells (Barranco et al., 2000). Wang et al. have modified the surface of core-shell down conversion nanoparticle (DCNPs) with 4-nitrophenol Cy7 (Nph) and amphiphilic distearyl phosphatidylethanolamine-polyethylene glycol (DSPE-PEG) (Wang et al., 2021b). Due to the intramolecular photoinduced electron transfer (PET) process, the Nph molecules coated on the surface of DCNPs have no fluorescence. In the presence of GSH, Nph reacted with GSH and formed Cy7-SG, and then the intramolecular PET process was activated and the fluorescence was recovered. Under the irradiation of 808 nm laser, Cy7-SG absorbed the incident light and transferred the excitation energy to Nd^3+^ of DCNP by means of nonradiative resonance energy transfer (NRET), resulting in sensitizing DCNPs produced an enhanced NIR-II fluorescence signal at 1550 nm (F_1550,808Ex_). In addition, 980 nm laser could not excite Cy7-SG molecules, but could directly excite DCNPs, so the fluorescence signal (F_1550,980Ex_) in the NIR-Ⅱ region of 1550 nm remained unchanged, resulting in a ratiometric fluorescence signal (F_1550,808Ex/_F_1550,980Ex_). The experimental results showed that there was a linear relationship between F_1550,808Ex_/F_1550,980Ex_ and GSH concentration. It realizes the accurate quantification and real-time imaging for GSH of colon cancer tissue *in situ* and played an important role in the early diagnosis of human diseases.

### Signal Molecules

It is well known that hydrogen sulfide (H_2_S) is the third important endogenous gas signal transduction compound after CO and NO (Szabo, 2007). It plays an important role in cell growth, cardiovascular protection, vasodilation, anti-inflammation, anti-oxidation, anti-apoptosis and other biological processes (Shi et al., 2018). The overexpression of cystathionine-β-synthase (CBS) in colon cancer cells promotes the production of H_2_S in tumor tissue (Szabo et al., 2013). Taking into account that the shortcomings of traditional detection methods, including colorimetry, surface-enhanced Raman scattering, electrochemical analysis and fluorescence analysis (Deng et al., 2019; Li et al., 2015; Wang et al., 2019c), which are limited to *in vitro* quantification. Wang et al. have developed an *in situ* H_2_S activable ratiometric nanoprobe with two NIR-II emission signals to detect H_2_S and intelligently illuminated colorectal cancer (Wang et al., 2021c). The nanoprobe consists of a down conversion nanoparticle (DCNP), which emitted NIR-II fluorescence at 1550 nm under the irradiation of a 980 nm laser (F_1550Em,980Ex_). Furthermore, human serum albumin (HSA) was combined with Ag^+^ on the surface of DCNP to form DCNP@HSA-Ag^+^ nanoprobe. Ag_2_S QDs were formed with the presence of H_2_S, which emitted fluorescence at approximately 1,050 nm on irradiation with an 808 nm laser (F_1050Em, 808Ex_) by an H_2_S-induced chemical reaction between H_2_S and Ag^+^. On the other hand, the fluorescence signal of DCNP was stable at 1550 nm (F_1550Em,980Ex_), resulting in the ratiometic signals (F_1050Em,808Ex_/F_1550Em 980Ex_) related to the H_2_S concentration. Therefore, the NIR-II ratiometric fluorescence nanoprobe could accurately quantify the detection of H_2_S *in* colon cancer through an endogenous H_2_S-induced *in situ* reduction reaction to form Ag_2_S QDs (Figure 6G). Recently, Xu et al. have also reported an activatable NIR-II fluorescent probe (NIR-II@Si) for the visualization of colorectal cancer (Xu et al., 2018). The activatable nanoprobes were comprised of two organic fluorophores: a rational designed boron-dipyrromethene (ZX-NIR) dye to generate the NIR-II emission only in the presence of H_2_S, and an aza-BODIPY (aza-BOD) that inerted to H_2_S, serving as the internal reference, which made with two steps: first, the two dyes ZX-NIR and aza-BOD were trapped into the hydrophobic interior of self-assembled micellar aggregate based on mPEG-DSPE; Second, *in situ* shell cross-linking with (N-trimethoxysilylpropyl-N,N,N-tri-n-butylammonium bromide) (TBNBr) to produce water-dispersible core-shell silica nanocomposites (NIR-II@Si) with a covalently cross-linked silica shell. By using this activatable and targeting specific probe for deep tissue imaging of H_2_S-rich colon cancer cells, colorectal cancers in animal models can be accurately identified.

### pH

**FIGURE 7:**
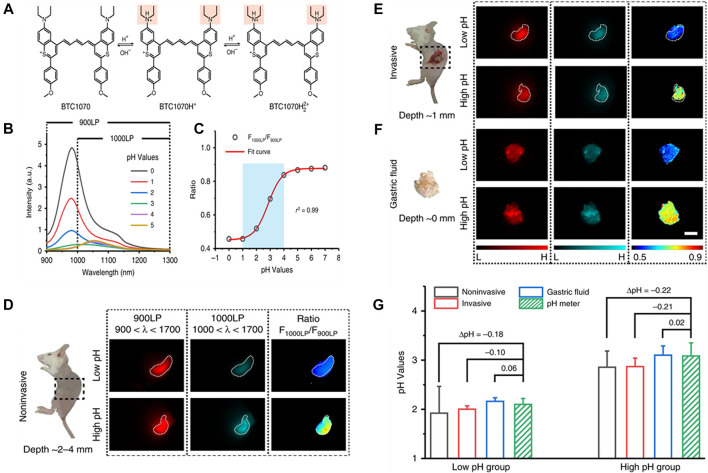
Protonation of BTC1070 and the corresponding spectra changes. **(A)** Protonation mechanism of NIR-II fluorophore BTC1070 showing stepwise protonation on nitrogen atoms. **(B)** The corresponding fluorescence spectra excited at 808 nm at various pH values. **(C)** Plot of fluorescence ratio changes as a function of pH values. Ratio = F_1000LP_/F_900LP_, F_1000LP_ and F_900LP_ denote the integrated intensity at wavelength of 1,000–1,300 nm and 900–1,300 nm, respectively. **(D–G)** *In vivo* ratiometric fluorescence imaging of gastric pH. **(D,F)** Left: digital photographs of mice and dissected stomach denote three imaging modes. **(D)** noninvasive imaging at ∼ 2–4 mm tissue depth; **(E)** invasive imaging of gastric fluid covered by ∼ 1 mm thickness of gastric wall; **(F)** imaging of exposed gastric fluid. **(G)** Comparison of pH values in mice stomach measured by ratiometric fluorescence imaging and standard pH meter. Data point with its error bar stands for mean ± *s*.d. derived from n = 3 biologically independent mice (reproduced from (Wang et al., 2019c) with permission from Springer Nature Publishing Group).

Recently, Wang et al. have developed a pH-responsive benzothiopyrylium pentamethine cyanine dye (BTC1070) for noninvasively quantitative measurement of gastric pH *in vivo* (Figure 7A) (Wang et al., 2019c). Because of their anti-quenching properties, these fluorophores showed spectral response to pH in aqueous solution. When pH changed from 5 to 0, the emission peak shifted from 1,065 to 980 nm under 808 nm excitation, and accompanied by a significant intensity change (Figure 7B). Plot of integrated intensity ratio from two wavelength regions (1000–1300 and 900–1300 nm) at pH 0–7.0 was well fitted by a sigmoidal equation (*R* ^2^ = 0.99), suggesting an optimal pH-sensitive range was 1.0–4.0 (Figure 7C). Based on the calibration results, the ability of BTC1070 to perform ratiometric imaging of gastric pH *in vivo* was further tested, mice were fed with simulated gastric juice with different pH values (pH1.3 and pH2.5) to simulate the pH environment of human stomach. As shown in Figure 7D, after intragastric administration of BTC1070 micellar solution, non-invasive ratio imaging could not only identify stomach profile from the left side of the abdomen with tissue depth of 2–4 mm, but also distinguish the two pH environments with clear pseudo-color contrast. Similar results were found in invasive ratiometric imaging of gastric juice wrapped in thin gastric wall (1 mm depth, Figure 7E) and exposed gastric juice imaging (0 mm depth, Figure 7F). The ratio was converted to pH value using calibration curve, which was consistent with the result measured by a standard pH meter (Figure 7G). BTC1070 can noninvasively detect gastric pH in a wide range of pH by a high contrast deep tissue ratiometric imaging, and achieve non-invasive gastric pH measurement at the depth of ∼4 mm, and its accuracy is reliable.

## NIR-II Ratiometric Photoacoustic Imaging for *In Vivo* Quantitative Analysis

Photoacoustic imaging (PAI) technology combines with the advantages of optical imaging and ultrasonic imaging to achieve high spatial resolution and good tissue penetration depth (∼cm depth) (Fu et al., 2019; Upputuri and Pramanik, 2019; Li et al., 2020) In particular, the photoacoustic imaging (PA) in NIR-II window has deeper tissue penetration depth and higher SBR(Zhou J. et al., 2018; Cai et al., 2019; Lyu et al., 2019). Therefore, NIR-II ratiometric PA is a suitable tool for *in vivo* quantitative analysis.

### ROS

**FIGURE 8:**
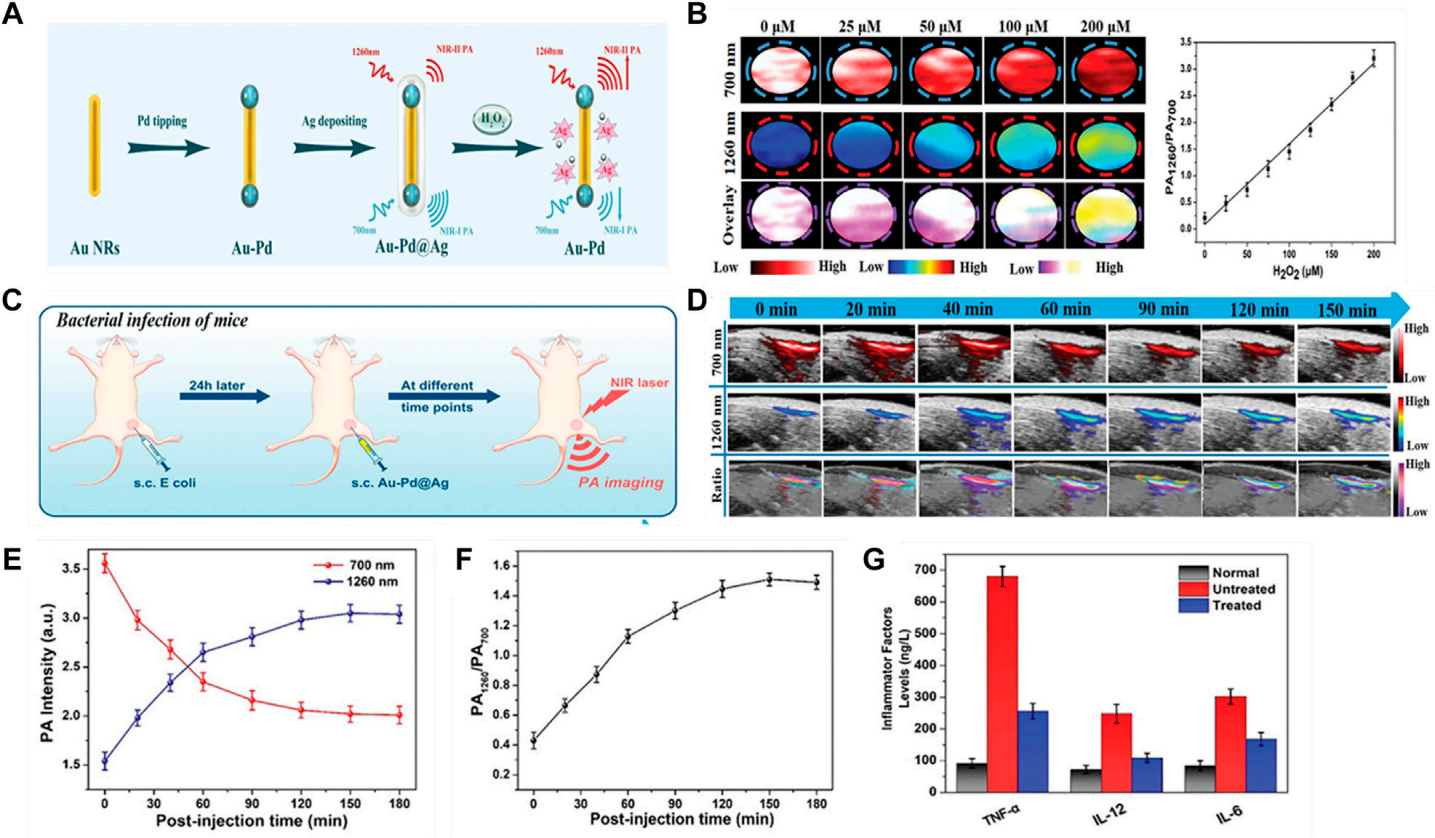
ROS-responsive ratiometric NIR PA imaging **(A)** Schematic illustration of the synthetic route of Au–Pd@Ag nanoprobe. **(B)** PA images at 700 and 1,260 nm of Au–Pd@Ag before and after incubated with different concentration of H_2_O_2_, and the ratiometric PA intensity (PA_1260_/PA_700_) as a function of the concentration of H_2_O_2_. **(C)** Schematic illustration showing detection of H_2_O_2_ in the inflamed region of mice infected subcutaneously with *E. coli* using the Au–Pd@Ag nanoprobe. **(D)** Representative PA images of the infected region before and after subcutaneous (s.c) injection of Au@Ag–Pd nanoprobe. **(E)** Time-dependent changes in PA_700_ and PA_1260_ intensities following Au–Pd@Ag injection. **(F)** Ratiometric PA_1260_/PA_700_ as a function of time in the above. **(G)** The level of inflammatory factors (including TNF-*a*, IL-12, IL-6) in the differentially treated animals (reproduced from (Ye et al., 2020) with permission from WILEY-VCH Verlag GmbH and Co. KGaA, Weinheim).

Ye et al. have reported a novel type of H_2_O_2_-responsive theranostic nanoplatform composed of Ag shells coated with Pd-tipped gold nanorods (Au-Pd@Ag NR) (Figure 8A) (Ye et al., 2020). The etching and oxidation of Ag shells by Ag^+^ ions released in the presence of H_2_O_2_, which effectively killed the bacteria in the body and triggered the absorption variation at 700 and 1260 nm. Ratiometric PA imaging (PA_1260_/PA_700_) at 1,260 and 700 nm accurately quantified H_2_O_2_ in models of bacterial infection, abdominal inflammation and osteoarthritis. Au-Pd@Ag nanoparticles have high PA signal (PA_700_) at 700 nm and relatively low signal (PA_1260_) at 1260 nm. Once exposure to H_2_O_2,_ Au-Pd@Ag triggered PA_700_ decrease and PA_1260_ increase in a concentration-dependent manner. In addition, there was a linear correlation between PA_1260_/PA_700_ ratio and H_2_O_2_ concentration (Figure 8B). They first analyzed whether Au-Pd@Ag nanoprobes can quantitatively detect endogenous H_2_O_2_ in *E. coli* mouse model by PAI modality (Figure 8C). Au-Pd@Ag nanoparticles were injected into the infected area 24 h later, PA_700_ and PA_1260_ signals were recorded in real time by LAZR PA Imaging System (Figure 8D). The signal intensity of PA_700_ in the infected area decreased in a time-dependent manner, at the same time, that of PA_1260_ increased along with time (Figure 8E). As a result, the PA_1260_/PA_700_ ratio in the inflammatory area gradually increased and peaked at 150 min post-injection with 3.5 times higher than the baseline (Figure 8F). In addition, further testing revealed that the PA_1260_/PA_700_ values remained unchanged in noninfected regions, but the level of inflammatory factors (including TNF-a, IL-12, IL-6) in the differentially treated animals were significantly decreased (Figure 8G). Therefore, Au-Pd@Ag nanoprobe can be used as a therapeutic platform to quantify H_2_O_2_ *in situ* and kills infiltrating bacteria as well as reduce inflammation.

### Metal Ions

**FIGURE 9:**
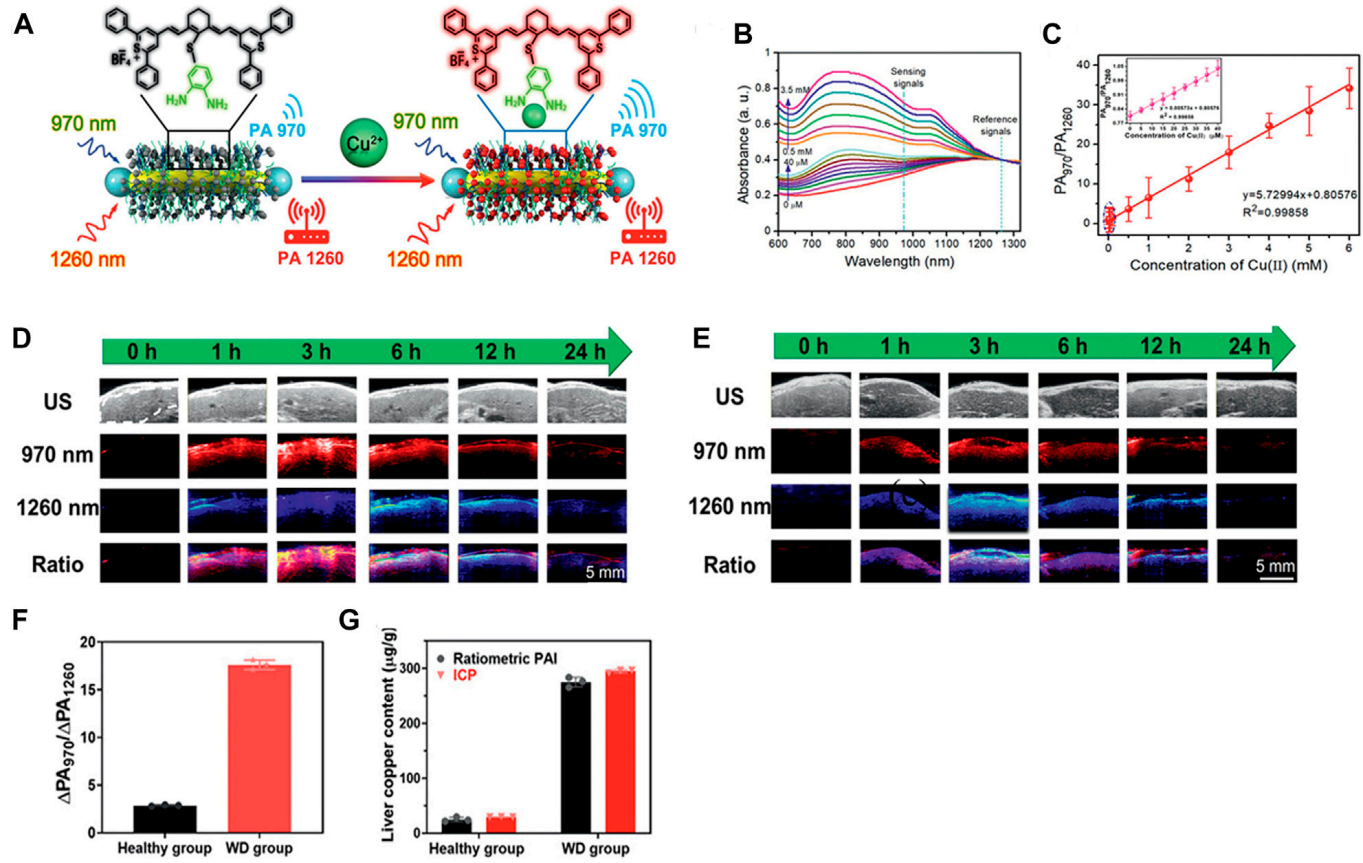
Cu^2+^-responsive ratiometric NIR PA imaging. **(A)** Illustration of design of Cu^2+^ -responsive ratiometric photoacoustic probe (AuNR-Pd@PIR970/PEG). **(B)** UV-vis spectrum of nanoprobe in different concentration of Cu^2+^. **(C)** PA_970_/PA_1260_ signal ratios of nanoprobe measured under various concentration of Cu^2+^. **(D)** Ultrasonic (B-mode) and PA images of mice liver with WD and **(E)** healthy mice. **(F)** Corresponding PA970/PA1260 value of WD and healthy group. **(G)** Liver Cu^2+^ contents measured by liver biopsy and ratiometric PA imaging of healthy and WD mice (reproduced from (Fu et al., 2021) with permission from Wiley-VCH GmbH).

NIR-II PA imaging can be used not only in the detection of ROS *in vivo*, but also in the quantitative analysis for disorders of metal metabolism, such as Cu^2+^ disorder in hereditary hepatolenticular degeneration, that is Wilson’s disease (WD). Excessive Cu^2+^in the liver can lead to a variety of liver diseases, such as liver injury and inflammation (Czlonkowska et al., 2018). Fu et al. have designed an activable ratiometric NIR-II PA imaging probe (AuNR-Pd@PIR970/PEG) (Fu et al., 2021). The AuNR-Pb@PIR970/PEG consisted of a NIR PA contrast agent named IR970, at 970 nm that selectively reacted with Cu^2+^, and the AuNR-Pd was with a strong absorption peak of 1,260 nm as a reference, which could be achieved the detection of Cu^2+^ by proportional PA (PA_970_/PA_1260_) (Figure 9A). As shown in Figure 9B, the absorption peak of AuNR-Pd@PIR970/PEG nanoprobe at 970 nm increased with the increase of Cu^2+^ concentration, while the absorption peak of AuNR-Pd at 1260 nm was almost unchanged. Subsequently, the PA imaging signals of the nanoprobes treated with different concentrations of Cu^2+^ at 970 and 1260 nm were recorded respectively (Figure 9C), the ratio of PA_970_/PA_1260_ increased significantly and had a good linear relationship with the concentration of Cu^2+^. The quantitative detection and visualization of Cu^2+^ in liver was feasible in mouse models of WD mice and healthy mice. The intensity of PA in WD mice at 970 nm (Δ PA_970_) and 1260 nm (ΔPA_1260_) were gradually increased until it reached a plateau at 4 h (Figure 9D). For healthy mice, the plateau period was reached within 2 h (Figure 9E), which was ascribed to the higher metabolic rate and better liver function of healthy mice than that of WD mice. The ΔPA_970_/ΔPA_1260_ in healthy mice was relied on the accumulation of probes in the liver (Figure 9F). WD mice were not only dominated by probes accumulation, but also by the activation of Cu^2+^ (Figure 9G). In general, the ratio PA detection method provides an accurate, rapid and simple non-invasive technique, and can be regarded as a promising tool for the early diagnosis of WD.

## NIR-II Fluorescence-Lifetime Imaging for *In Vivo* Quantitative Analysis

Fluorescence lifetime imaging is a time-resolved measurement technique for analysis and imaging by collecting different fluorescence lifetime signals. Moreover, the fluorescence lifetime is not affected by excited light power and tissue penetration depth, and it does not need to be calibrated at different depths, thereby it is often used as a quantitative probe, which lays a foundation for multi-coding quantitative detection of molecules or biomarkers *in vitro* and *in vivo* (Sarder et al., 2015). For example, the rare Earth fluorescence lifetime probe has a great application prospect in the fields of biological imaging, biosensor, multi-channel composite imaging and high-throughput detection analysis (Johnson et al., 2017; Zhou L. et al., 2018; Fan et al., 2018; Kong et al., 2019; Tan et al., 2020; Zhao et al., 2020).

### ROS

**FIGURE 10:**
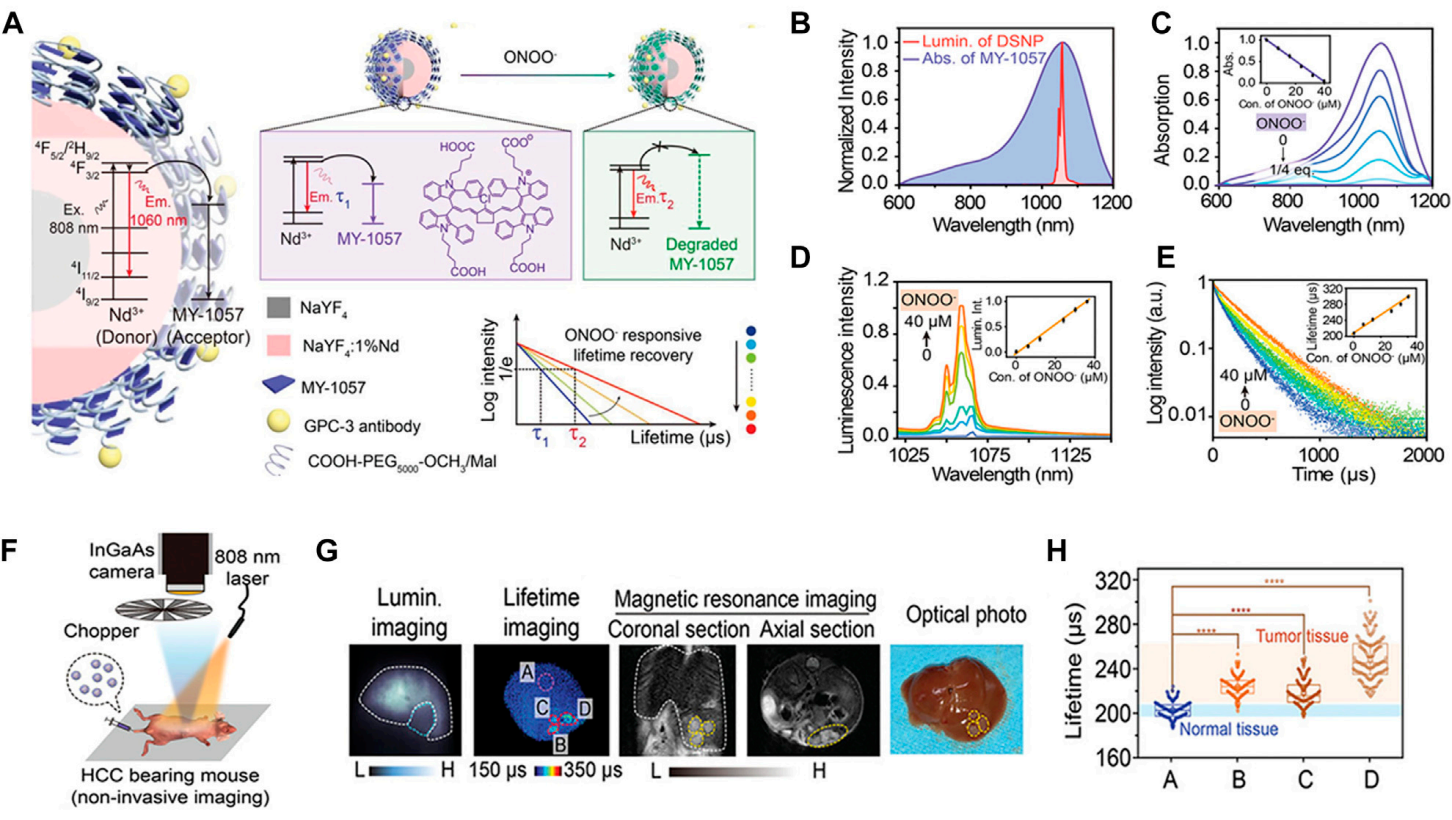
Lifetime-resolved imaging for ONOO^−^ dectection. **(A)** Schematic illustration of the ONOO^−^-responsive nanosensor DSNP@MY-1057-GPC-3. In the presence of ONOO^−^, the structure of the energy acceptor MY-1057 degrades sensitively, leading to the lifetime recovery in NIR-II region. **(B)** Overlap of the MY-1057 absorption and DSNP luminescence emission spectra. **(C)** Absorption of MY-1057 as a function of ONOO^−^ (0–1/4 equiv.). **(D)** Luminescence emission intensity and **(e)** lifetime response of DSNP@MY-1057-GPC-3 at 1,060 nm as a function ONOO^−^ concentration. **(F)** *In situ* HCC tumor detection by noninvasive imaging setup after administration of DSNP@MY-1057-GPC-3 nanosensor. **(G)** Noninvasive intensity-based imaging, lifetime-based imaging of a mouse bearing multiple HCC lesions and optical photo of the dissected liver. ROI A: normal hepatic tissue; ROI B–D: tumor lesions distinguished from lifetime imaging. **(H)** Lifetime extracted from ROI A–D of **(G)** (reproduced from (Zhao et al., 2020) with permission from WILEY-VCH Verlag GmbH and Co., KGaA, Weinheim).

Recently, Zhao et al. have synthesized a new type of TME response NIR-II fluorescence nanosensor (NaYF_4_@NaYF_4_:1%Nd^3+^-MY1057), in which Nd^3+^ absorbed the excited light energy of 808 nm and produced NIR-II fluorescence of 1060 nm (Zhao et al., 2020). In response to ONOO^−^ at tumor, the structure of energy acceptor MY-1057 degrades, resulting in the lifetime recovery of nanosensor (Figure 10A). In order to obtain high luminous intensity and stable lifetime of *in vivo* imaging, lanthanide downshift nanoparticles (DSNPs) with β-NaYF_4_@NaYF_4_:1%Nd as NIR-II FRET donor with 1060 nm emission were synthesized (Figure 10B). The absorption of MY-1057 at 1057 nm gradually decreased with ONOO^−^ treatment due to structural degradation (Figure 10C), accompanied by the nanosensor’s luminous intensity recovery (Figure 10D), and along with lifetime of the nanosensor recovered from 203 ± 2 μs to 298 ± 2 μs (Figure 10E). Then, the humanized mouse HCC model was intravenously injected with DSNP@MY1057-GPC-3 nanosensor (15 mg/kg) (Figure 10F). Three tumor lesions with a survival time of 215 ± 27–249 ± 43 μs and the normal liver tissue of 204 ± 10 μs were accurately distinguished, respectively (Figures 10G,H). The novel composite probe opens a new way for the specific response of ultra-precision cancer diagnosis, and provides a new idea for the quantitative study of pathological parameters and real-time dynamic imaging *in vivo*.

### Biomarkers

**FIGURE 11:**
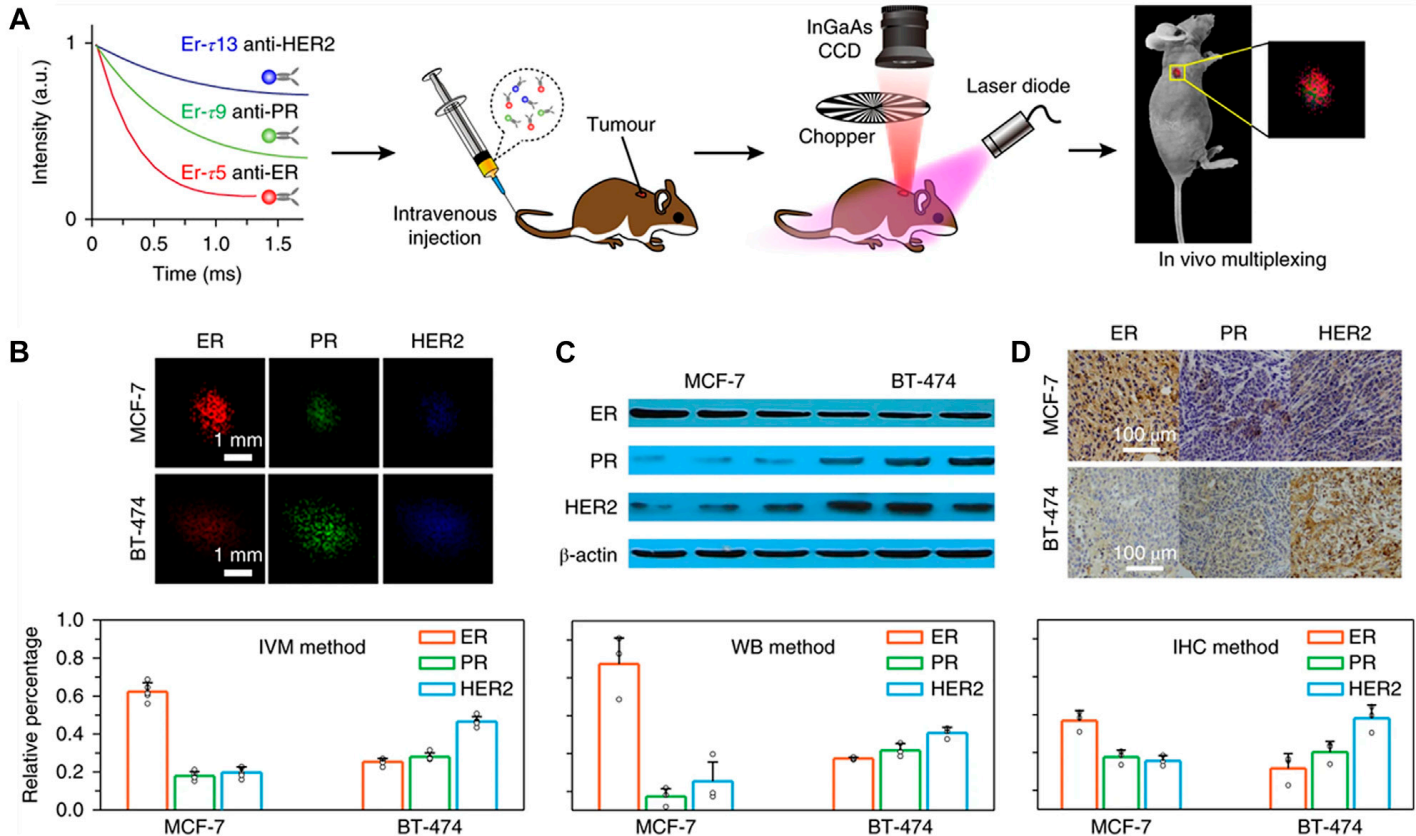
Lifetime-resolved imaging for *in vivo* multiplexing (IVM) of tumor biomarkers. **(A)** Schematics illustrating animal experiment procedures. Three batches of Er nanoparticles exhibiting distinct lifetimes are conjugated to three antibodies (anti-ER, anti-PR and anti-HER2), respectively, and intravenously injected into the mouse *via* tail vein. Lifetime-resolved imaging is then performed to quantify the biomarker expressions on the tumor *via* IVM. CCD, charge-coupled device. **(B)** Lifetime-resolved images for the MCF-7 and BT-474 tumors are decomposed into the three lifetime channels, represented by the red, green and blue monochromatic image sets. **(C,D)** *In vitro* western blot **(C)** and *ex vivo* immunohistochemistry assay **(D)** results and calculated biomarker expression patterns of the two tumors subtypes IVM: *in vivo* multiplexing; WB: western blot; IHC: immunohistochemistry (reproduced from (Fan et al., 2018) with permission from Springer Nature Publishing Group).

In 2018, Fan et al. have synthesized a NIR-II fluorescence-lifetime nanoparticles doped with lanthanides that feasibly designed the luminescence time for quantitative analysis *in vivo* imaging with multiplexing in the time domain (Fan et al., 2018). Taking the fluorescence probe doped with Er as an example, the fluorescence lifetime control across three orders (μs-ms) of magnitude were realized by adjusting the thickness of the second energy transfer layer and the doping amount of Er^3+^. In this fluorescence mode, the lifetime remained unchanged even if the SNR was less than 1.5, the fluorescence lifetime of the fluorescence probe has nothing to do with the tissue depth, so it can be used for quantitative researches without calibration at different depths. In nude mice models with bearing xenografts of MCF-7or BT-474 cells, the biomarker expressions of the tumor subtypes were quantified by resolving the three lifetime components simultaneously with a pattern recognition algorithm (Niehorster et al., 2016) (Figure 11A). It was worth noting that the expression patterns of the three biomarkers in different tumors were obviously different for the two tumor subtypes (Figure 11B). MCF-7 tumors expressed a large number of ER (62.3%), PR (17.9%) and HER2 (19.8%) at moderate levels. In BT-474 tumors, the expression rate of HER2 was the highest (46.6%), followed by PR (28%) and ER (25.4%). These expression patterns obtained by *in vivo* multiplexing (IVM) method were highly consistent with the Western blot (WB) results (Figure 11C). The IVM method was also compared with the traditional *in vitro* immunohistochemical (IHC) method. Three mice were used by IHC for each tumor subtype, and the images were analyzed with IHC (Figure 11D). There was a good correlation between the two methods for the expression patterns of biomarkers in the two tumor subtypes. Traditional IHC examines only one biomarker per tissue section. In contrast, IVM allows simultaneous quantification of all biomarkers to minimize uncertainty caused by biopsies, sample processing and scoring processes.

## Perspectives and Challenges

Overall, NIR-II bioimaging have studied in the past decades for various *in vivo* quantitative applications, such as NIR-II fluorescence, PA and luminescence lifetime imaging for *in vivo* quantitative analysis, which not only influences fundamental biomedical research studies in NIR-II probes and NIR-II imaging technologies, but also presents a great potential for clinical translational researches in the future. However, there are some challenges need to be overcome to expand the NIR-II *in vivo* quantitative imaging from its current stage to a higher level, which could help researchers to further understand the complex systems and pathways of living organisms. In the following points, we envisage the main concerns regarding NIR-II probes and imaging techniques, and give some advices and prospects for NIR-II *in vivo* quantitative analyses in future biomedical applications.

First, making *in vivo* quantification more comprehensive and accurate. Due to the integral inhomogeneity caused by the complexity of the tissue structure, and the current quantitative analyses are mostly based on the 2D plane of fluorescence imaging. Therefore, it is difficult to achieve “a glimpse to know the whole panther” based on a single 2D plane of imaging results. It is still facing challenges to acquire the regional morphological quantitation and total moles of substance. With the 3D scanning function of NIR-II imaging technology can make more accurate morphological quantitative information. The application of the NIR-II laser confocal and the 3D tissue scanning function of the light sheet intravital microscope to the *in vivo* quantification can solve aforementioned problems to a certain extent. Nevertheless, the scanning speed and the data processing speed of the computer are still facing challenges. The application of 3D scanning functional NIR-II imaging technology provides more comprehensive and accurate quantitative information for living tissue morphology. For example, NIR-II confocal and light-sheet microscopy have shown corresponding advantages in 3D imaging of living tissues (Zhu et al., 2018b; Wang F. et al., 2019; Wang F. et al., 2021), but the higher scanning speed and the huger data processing required for *in vivo* quantification still are troubles to be settled urgently.

Second, simultaneously acquiring more different quantitative properties. Due to the complexity and dynamic characteristics of organisms, static single-source signal acquisition cannot give a complete overview of physiological and pathological changes. Therefore, the multi-channel imaging techniques and multi-response probes should be developed for NIR-II *in vivo* quantitative analysis. Multi-response NIR-II probes can respond to different analytes and have differentiable signals for various analytes, and the real-time multi-channel NIR-II imaging techniques can simultaneously record multiple events (Fan et al., 2018; Cao J. et al., 2019; Wang T. et al., 2021; Shinn et al., 2021), providing more *in vivo* quantitative properties for understanding the physiological and pathological processes of living bodies.

Last but not least, the application and development of NIR-II *in vivo* quantitative researches can draw lessons from the experience of traditional UV-Vis-NIR techniques, but it will also face the same challenges as traditional techniques, which is mainly limited by the lack of suitable NIR-II probes. Up to now, there are no FDA-approved NIR-II probes available for clinical use, so it is a challenge to realize NIR-II *in vivo* quantitative researches in clinical translation. NIR-II ratiometric and lifetime probes have showed excellent performance in *in vivo* quantification. However, due to the lack of superior biocompatible probes, the current reported studies mostly use inorganic materials, such as QDs [Ag_2_S(Chen et al., 2018)], noble metals [Au-Pb (Ye et al., 2020)], and rare-earth [NaEr_x_Y_1_-_x_F_4_@NaYF_4_ (Wang F. et al., 2019)], that possess potential biological toxicity. Following the rule that “taken from nature, used in nature”, obtaining probe materials with NIR-II optical properties from nature of biological source is a desirable approach to retrieve biocompatible probes (Saif et al., 2020; Shang et al., 2020). More importantly, it is worth drawing on the successful experience of FDA-approved NIR molecular probes indocyanine green (ICG) and methylene blue (MB) (Polom et al., 2014; Carr et al., 2018), committing to prepare NIR-II molecular probes. The application range of NIR-II *in vivo* quantitative researches is also limited by NIR-II probes. Currently, there is a lack of high-performance NIR-II probes for quantitative analyses in *in vivo* of gene expressions and neuroscience researches. *In situ* visualization of gene expressions in living bodies requires the development of genetically encoded fluorescent proteins with NIR-II longer emission wavelengths in NIR-II region. This would require to identify a NIR-II fluorescent protein, and to engineer the protein structures as well as to identify the encoding gene that are responsible for the NIR-II fluorescence emission in the bacteria, contributing to further study the expression of NIR-II fluorescent proteins in mammalian cells (Qian et al., 2019; Shen et al., 2020). The membrane potential-sensitive NIR-II probes may offer the possibility to simultaneously monitor neural activities of large numbers of nerve cell populations in living tissues (Liu et al., 2020d; Shemetov et al., 2021). This would require to the use of activable NIR-II probes that can specifically sense bio-voltage changes, ions (Ca^2+^, K^+^) and neurotransmitters (dopamine).
